# Randomly controlled drivers using minimally invasive sampling: assessment of drug prevalence in Western Switzerland over two time periods

**DOI:** 10.1186/s12889-022-14883-2

**Published:** 2022-12-28

**Authors:** Timothée Joye, Julien Déglon, Nicolas Donzé, Federica Gilardi, Jonathan Sidibé, Bernard Favrat, Marc Augsburger, Aurélien Thomas

**Affiliations:** 1 Forensic Toxicology and Chemistry Unit, University Center of Legal Medicine Lausanne-Geneva, Lausanne University Hospital, Geneva University Hospital, Chemin de la Vulliette 4, Lausanne 25, 1000 Switzerland; 2 Clinical Chemistry and Toxicology Unit, Valais Hospital, Avenue du Grand Champsec 86, Sion, 1950 Switzerland; 3 Faculty Unit of Toxicology, University Center of Legal Medicine Lausanne-Geneva, Faculty of Biology and Medicine, University of Lausanne, Chemin de la Vulliette 4, Lausanne 25, 1000 Switzerland; 4 Unit of traffic medicine and traffic psychology, University Center of Legal Medicine Lausanne-Geneva, Lausanne University Hospital, Geneva University Hospital, Rue Saint-Martin 26, Lausanne, 1005 Switzerland; 5 Centre for Primary Care and Public Health (Unisanté), University of Lausanne, Rue du Bugnon 44, Lausanne, 1011 Switzerland

**Keywords:** Driving under the influence of drugs, Prevention, Psychoactive substances, Roadside controls, Minimally invasive sampling, Oral fluids, Dried blood spots

## Abstract

**Background:**

According to the World Health Organization, road traffic injuries lead to 1.3 million deaths each year and represent the leading cause of death for young adults under 30 years old. The use of psychoactive substances, including alcohol, drugs and pharmaceuticals, is a well-known risk factor for road traffic injuries. Our study aims to assess the prevalence of substances consumed by drivers in western Switzerland. Such studies are pivotal to improving prevention and developing public awareness campaigns.

**Methods:**

To assess the prevalence of psychoactive substances among drivers, roadside controls were performed in collaboration with local police, using their classical sampling procedures to detect drivers under the influence of drugs or alcohol over two time periods (P1: 2006-2008, P2: 2017-2020). When impaired driving was not suspected by the police, minimally invasive sampling strategies (i.e., oral fluids during P1 and dried blood spots during P2) were performed on volunteer drivers after a road safety survey. A posteriori analyses and statistical interpretation were then performed*.*

**Results:**

Among the 1605 drivers included in the study, 1048 volunteers provided an oral fluid sample, while 299 provided a dried blood spot sample. The percentage of drivers testing positive for at least one substance that can impact driving abilities was stable over time, with a rate of 10.5% positivity measured over both periods. Considering the different categories of substances, a slight variation was observed between both periods, with 7.6 and 6.3% of pharmaceuticals and 3.6 and 4.9% of illicit drugs for P1 and P2, respectively. Regarding the consumption of illicit drugs, the highest percentage of positivity was measured in biological fluids of drivers under the age of 35, during nights and week-ends, periods which are considered particularly prone to fatal accidents for this age group. Disturbingly, the road safety survey highlighted that drivers’ perception of the risk of getting positively controlled while driving after drug consumption is low (3.3 on a 1-to-10 scale, *N* = 299).

**Conclusion:**

The number of positive cases measured in voluntary drivers who passed the preliminary police check demonstrates the importance of systematic biofluid sampling strategies regarding driving under the influence of psychoactive substances. Although the number of fatal road accidents globally has decreased over time, the results of this study reveal the need for both better prevention and deterrent processes that could potentially reduce the risk of fatal road accidents associated with drug consumption.

**Supplementary Information:**

The online version contains supplementary material available at 10.1186/s12889-022-14883-2.

## Background

Road accidents represent an important cause of deaths and injuries, and their prevention must be a priority. According to the World Health Organization, 1.35 million people died, and approximately 50 million people were injured from road traffic accidents worldwide in 2018 [1]. One of the major causes of road accidents, particularly in Western countries, is the impairment of driving capabilities caused by the effect of psychotropic substances, drugs, and alcohol [2, 3]. For instance, survey-based self-reported data suggest that in the US, 27.7 million people drove under the influence of alcohol and 10.1 million drove under the influence of illicit drugs in 2014 [4]. Nevertheless, prevention has improved the situation in previous years, especially in Western countries. For instance, according to the Federal Statistical Office (OSF), in Switzerland, the number of victims of fatal accidents has significantly decreased in the last 26 years. Nevertheless, considering relative values, impaired drivers (either due to alcohol, illicit drugs or pharmaceuticals) were still responsible for a more or less constant percentage (16 to 25%) of fatal accidents between 1995 and 2018 [5, 6]. In addition, the prevalence of psychoactive drugs within the global Swiss population is constantly increasing. Reports from the Swiss Federal Statistical Office show that 50% of the population aged above 15 years old were taking a pharmaceutical every week in 2017, compared to 38% in 1992 [7]. The proportion of individuals taking pharmaceuticals increases with age, with 84% of the population aged over 75 years consuming pharmaceuticals weekly. In 2020, psychotropic drugs were the most prescribed drugs in Switzerland, representing 22.7% of all pharmaceuticals according to the Swiss Health Observatory [8]. Within this category, antidepressants were the most prescribed drug. In 2018, the extrapolated one-year prevalence of benzodiazepines and z-drugs for the general Swiss population reached 10.5% [9]. Switzerland is also one of the largest opioid and opiate consumer in the world, with prescriptions increasing by an average of 12% per year between 1985 and 2015 [10]. Alcohol is one of the most widespread psychoactive substances, with 10.9% of the Swiss population reporting daily consumption and 15.9% reporting intermittent drunkenness. Regarding illicit drugs, in a study based on a self-report survey conducted in 2016, 7.3% of the population declared cannabis consumption within the last year, and 3.1% declared cannabis consumption within the last month. For cocaine, these values reached 0.7 and 0.1%, respectively [11]. As individuals tend to underreport illicit and stigmatizing behaviours, these figures likely underestimate the actual prevalence of illicit drugs, particularly the consumption of cocaine. Furthermore, high frequency consumers of illicit drugs can be more difficult to reach using surveys, as they are often marginalised [12]. Thus, the drug-consumer population is likely underrepresented in self-report surveys [11].

The risk evaluation of driving under the influence of psychoactive substances is not an easy task. In many countries, including Switzerland, zero tolerance is applied concerning drivers towards classic illicit drugs [13–15]. Nevertheless, the situation is much more complex regarding the toxicological interpretation of pharmaceuticals leading to driving impairments [16]. Indeed, if many pharmaceuticals might lead to an enhancement of the risk of accident, the diversity of chemical properties and pharmacological effects do not allow a general statement on the driving abilities [17]. Moreover, many people taking pharmaceuticals that could impair driving are not aware of the risks [18]. According to Swiss law, the driving capability under these substances is determined by “a three pillars expertise”, including police assessment, medical expertise and toxicological analysis in blood [15]. In Europe, the project Driving Under Influence of Drugs, Alcohol and Medicines (DRUID) showed a willingness to harmonise the classification of pharmaceuticals by providing scientific-based recommendations to driving under the influence of psychoactive substance issues [19]. Therefore, to increase the understanding of Driving Under the Influence of Drugs (DUID) cases and improve both prevention and deterrent measures, a better understanding of the prevalence of the consumed substances and their toxicological effects is of interest.

Previous studies have shown significant differences in terms of substance distribution between samples collected from suspected drug-impaired drivers [15, 20–23] and those collected in random approaches [24–26]. Indeed, consumption habits are in constant evolution, and new substances, including new psychoactive substances (NPS), are arriving on the market. The variety of pharmacological effects complicates the identification of psychoactive substance consumption during preliminary on-site controls. Therefore, performing systematic sample collection and thus developing on-site, simple, and non-invasive sampling strategies for unbiased sample collection and more accurate knowledge of the prevalence of the consumed substances appears as an appropriate approach. Among those strategies, on-site collection of oral fluid (OF) has been indicated as a potential tool to evaluate DUID at roadside traffic controls [27]. Dried blood spots (DBS) also provide an interesting alternative since only a limited volume of blood (10-20 μL) can be collected by either finger or heel pricking. The blood is adsorbed and dried on a solid phase (cellulose), and sample collection requires minimal training that can easily be performed in a nonmedical environment [28–30].

This study aims to present the prevalence of driving under the influence of drugs in western Switzerland between 2006 and 2008 and 2017-2020. This study also provides insight into drivers’ perception of roadside controls and the bias associated with the current suspicion-based sampling procedures. The interest in the systematic use of on-site OF and DBS microsampling devices used by minimally trained technicians in a nonmedical environment is also discussed.

## Methods

### Study design

**Fig. 1:**
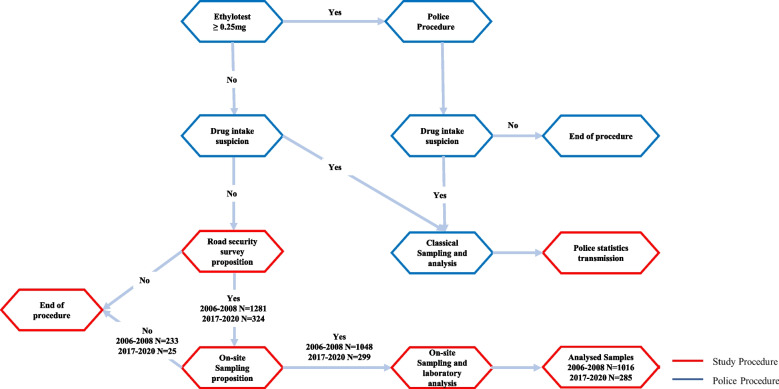
Design of both periods of the study (P1: 2006-2008; P2: 2017-2020)

Both parts of the study were performed according to the same general protocol in collaboration with local police forces in Western Switzerland (Canton of Vaud, Valais and Fribourg) during 31 controls. All drivers were systematically stopped without any suspicion, and the police officers proceeded with their classical procedure (Fig. 1). Alcohol consumption could be measured on a systematic basis on all drivers using breath testing (breath alcohol concentration, BrAC), while drug testing was only performed in cases of preliminary suspicion. If the drivers were either alcohol positive (BrAC > 0.25 mg/L) or if drug consumption was suspected, classical sampling and analysis were performed by the police assisted by medical staff. The anonymous statistics regarding those samples were sent by the police after analysis. When no consumption was suspected by the police, the drivers were directed towards a second checkpoint where a research associate introduced the study to the drivers and asked them if they wanted to participate in the study. In the first instance, the participants who had given their consent were asked to answer a road security survey with the goal of introducing them to the study and informing them of the sampling procedure in parallel with collecting insightful data. In a second instance, the participants were asked to provide a biological sample. During the first part of the study, between 2006 and 2008, the collected biological samples were OF, while during the second part occurring between 2017 and 2020, DBS were collected as described below. The samples were then anonymously analysed in the laboratory in a third instance. All analytical procedures were validated according to the Food and Drug Administration (FDA) guidelines and certified by the ISO/CEI 17025 standard. The police did not take part in any steps of the study procedure (Fig. 1).

### Biological sample collection and analysis

#### OF sampling and LC–MS/MS analyses from 2006 to 2008

OF samples were collected using a Salivette® (Sarstedt®). The OF collection swab was placed in the participant’s mouth and chewed for approximately 60 seconds to stimulate salivation. Then, the swab with the absorbed saliva was placed in the Salivette tube. Once in the lab, the samples were centrifuged for 2 minutes to separate the saliva from the swab, particles and mucus. The samples were then kept at − 20 °C until analysis. A double extraction process was performed on the OF swabs using a 1:1 mixture of methanol/acetonitrile to ensure that no substances were trapped in the cotton swabs. After extraction of the methanol/acetonitrile mix, the samples were analysed by LC–MS/MS (Shimadzu LC-20 Prominence and AB Sciex 3200 Q trap). The method targeted a total of 107 substances in multiple reaction monitoring mode (MRM), including 103 drugs of abuse and pharmaceuticals that are reported to affect driving capability. Among these substances, caffeine, nicotine, cotinine and paracetamol were only used as analytical references to ensure the quality of the analytical process. In parallel, ethanol was measured using headspace (HS) gas chromatography (GC) coupled with a flame ionization detector (FID).

### DBS sampling and LC-HRMS analyses during the 2017-2020 period

Blood drops were produced by finger pricking using a single use lancet (BD Microtrainer Contact-Activated Lancet®) after disinfection. The first blood drop was removed, and the second was collected by capillarity using a HemaXis™ device (DBS System). Using this protocol, one to four 10 μL DBSs were collected, and samples were then separately placed in a Minigrip® bag and kept at room temperature prior to the analysis.

A double extraction process on DBS was performed using a published procedure for large-scale screening of drugs and pharmaceuticals containing over 1065 substances [31]. Despite the technological evolution and the analytical advantages provided by this approach, the results presented hereafter focus on the same 103 substances as during the 2006-2008 period (Supplementary Table 1). Unfortunately, alcohol consumption could not be evaluated using DBS analysis.

### Data treatment

After direct on-site anonymization and randomization, questionnaire data were associated with laboratory analyses using a reference number. Questionnaires based on a previous Canadian study included general information regarding the type of vehicle, the drivers’ sex and age [25]. Questions concerning the drivers’ perception of the probability of being controlled after either alcohol or drug consumption were also asked during the second time period. Analytical results were separated depending on the type of road (city, highways, and main roads). All days of the week were included in the study and categorized as week (Monday to Friday 8 pm.) or weekend (Friday 8 pm. to Monday 4 am.) and separated into 3 time periods (4 am-12 pm “Morning”, 12 pm-8 pm “Afternoon” and 8 pm-4 am “Night”).

## Results

### Characteristics of the drivers

During the 24 controls carried out using OF during the first step of the study, 1048 out of 1281 (82%) participants agreed to provide a saliva sample, of which 1016 were analysed (Fig. 1). Among those participants, 70% were men, and the average age was 41 ± 15 (ranging from 16 to 90) years old. During the second step of the study, 324 drivers agreed to participate, among which 299 (92%) accepted blood sampling by finger pricking, showing a slight increase compared with the 2006-2008 study (Table 1). Among the participants in this second stage of the study, the average age was 39 ± 15 (ranging from 16 to 78), and 65% were men. These results are in agreement with published statistics acquired in 2015 in Switzerland, where 40% of drivers are women (60% for men) [32]. As expected, people positive for pharmaceuticals were slightly older than the average age of participants, while people positive for an illicit substance were younger (Table 1) [33]. Overall, similar results and tendencies were observed for the two periods covered by the study.

**Table 1:** Descriptive results regarding the study participants

	OF samples 2006-2008 (*N* = 1281)	DBS samples 2017-2020 (*N* = 324)
Female/Male drivers	30%/70%	35%/65%
Cars	97%	93%
Sampling acceptation	82%	92%
Prevalence of positive samples	10.5%	10.5%
Average age (years)	41 ± 15 (16-90)	39 ± 15 (16-78)
Average age among drivers positive for pharmaceuticals	48 ± 16 (18-80)	44 ± 14 (20-66)
Average age among drivers positive for illicit drugs	34 ± 11 (19-61)	33 ± 11 (20-59)
Perception of the probability of being controlled after alcohol consumption (1 to 10)	–	3.8 ± 2.3
Perception of the probability of being controlled after illicit drug consumption (1 to 10)	–	3.3 ± 2.3

Interestingly, during the second period of the study, when drivers were asked about their perception of the probability (on a scale of 1 being unlikely to 10 being very probable) of getting controlled by the police after either excessive alcohol (BrAC > 0.25 mg/L) or illicit drug consumption, the average answers were 3.8 ± 2.3 and 3.3 ± 2.3, respectively.

### Prevalence of drug consumption between 2006 and 2008

**Fig. 2:**
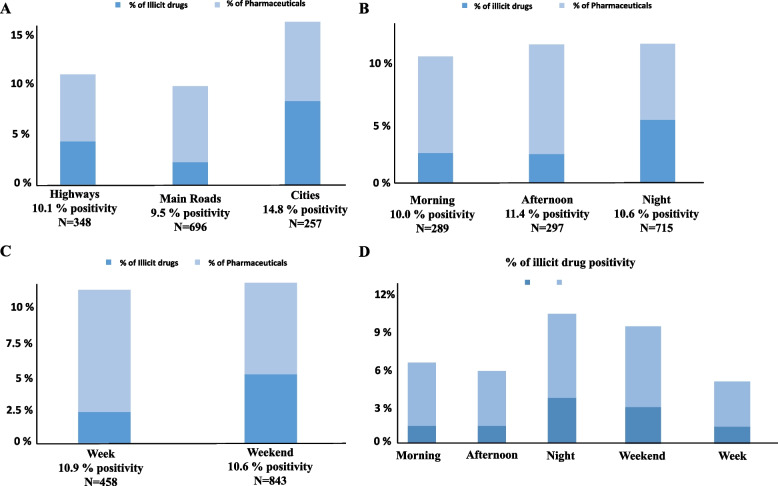
Participants positivity for at least one substance impairing driving ability. Comparison based on the road type (**A**) and the time period within the day (**B**) and week (**C**). The time period comparison for young drivers (< 35 years old) is presented in Panel (**D**) regarding illicit drugs. N.b.: one driver can be positive for both illicit and pharmaceuticals at the same time

**Fig. 3:**
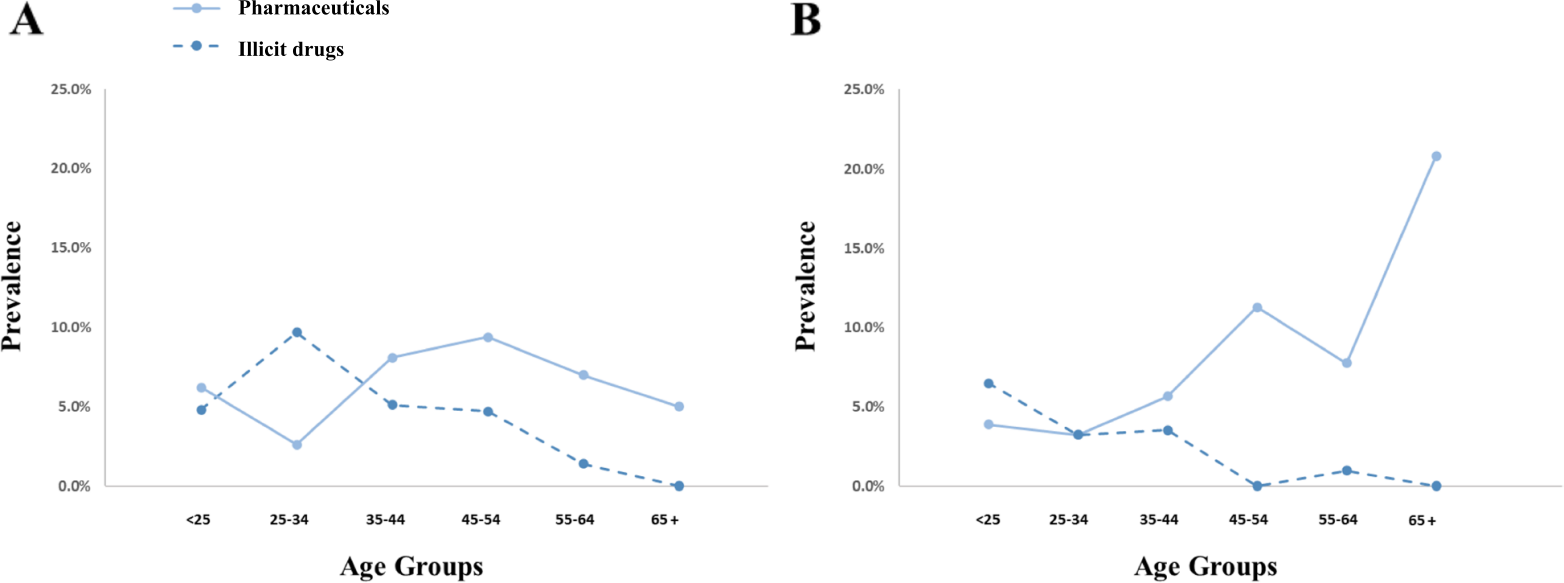
Age group comparison for illicit and pharmaceuticals during nights and weekends (**A**) and the other time periods (**B**). The continuous line represents the pharmaceuticals, while the dotted line represents the illicit drugs

For OF samples collected during the 2006-2008 period, analyses were performed on a list of 103 substances, including benzodiazepines, antidepressants, amphetamines, barbiturates, antipsychotics, opioids, cocaine, cannabinoids, and a few other substances considered as “other drugs” (Supplementary Table 1). Among the 1048 collected samples, 32 were not analysed due to technical reasons either during the sampling or the analytical procedure. Considering the 1016 analysed samples, 10.5% (107) were positive for a substance that could impact driving abilities (according to the DRUID 6th framework classification programme cofounded by the European Commission [34]). A total of 7.6% (77) of the samples were positive for a pharmaceutical, while 3.6% (37) were positive for an illicit drug (N.b.: one driver can be positive for both illicit and pharmaceuticals at the same time). Based on the OF sample analyses, 35 participants (3.4%) were positive for ethanol in concentrations below the legal threshold after being tested by the police (BrAc < 0.25 mg/L). Among the pharmaceuticals, the most frequently detected class of molecules was antidepressants, which were present in 3.3% of the cases, while benzodiazepines and opioids were present in 2.9 and 1.6% of the cases, respectively. Regarding drugs of abuse, cocaine was the most consumed substance, being present in 3.0% of the 1016 analysed samples, followed by cannabinoids (0.6%). Considering both periods of the study, data analysis was performed based on the type of roads and the time period within the day and the week (Fig. 2). The number of positive cases of pharmaceuticals was more or less the same when comparing samples collected on highways, main roads and urban areas (6.6, 7.5 and 7.8%, respectively). However, important differences were observed regarding drugs of abuse (4.3, 2.2 and 8.2%, respectively (Fig. 2A)). When considering the period within the week, an increase in illicit drugs and a decrease in pharmaceutical consumption were observed during weekends (Fig. 2C). Considering the time of the day, the consumption of illicit drugs was more pronounced during the night, whereas an opposite trend was observed for pharmaceuticals (Fig. 2B). Interestingly, the tendency showing an increase in illicit drug consumption during the night and weekends was exacerbated for younger drivers (< 35 years old) (Fig. 2D). These results confirm weekend and night periods to be particularly at-risk time periods, and drivers under the age of 35 are the population most prone to consume an illicit drug (Fig. 3). Conversely, and as expected, the prevalence of pharmaceuticals tends to increase within the older groups.

During the 2006-2008 period, the police collected 19 blood samples of people suspected of driving under the effect of psychoactive substances. Among these samples, 14 (74%) were positive for ethanol, with a blood concentration over 0.8 g/kg. The five other drivers were positive for cannabinoids above the legal threshold (THC > 1.5 ng/ml). Moreover, one driver positive for THC was also positive for methamphetamine, while 3 drivers positive for alcohol were also positive for cocaine, methadone and fluoxetine. Interestingly, the consumption of opioids, cocaine and amphetamines was not suspected by the police in these cases.

### Prevalence of drug consumption between 2017 and 2020

**Fig. 4:**
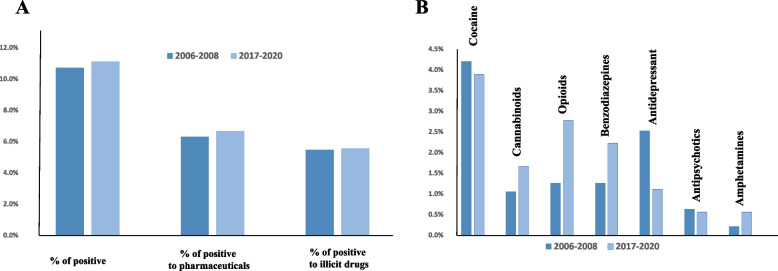
Comparison of positive cases (**A**) and percentage of occurrence of the different classes of substances (**B**) between 2006 and 2008 and 2017-2020, focusing on nights and weekends

During the second part of the study, 14 out of the 299 collected samples were not analysed for technical reasons. Among the 285 analysed samples, the results were similar to those obtained between 2006 and 2008, with 30 (10.5%) positive samples, including 18 (6.3%) and 14 (4.9%) positive for pharmaceuticals and illicit drugs, respectively. In addition, the same classes of molecules detected with OF fluid collection were highly represented. Indeed, benzodiazepines were present in 2.8% of the cases, followed by opioids and antidepressants being present in 1.8 and 0.7% of the cases, respectively. Regarding illicit drugs, the same tendency can be observed, with cocaine being present in 2.8% of the cases and cannabinoids in 2.1%. Interestingly, both the proportion of drivers positive for drug consumption and the classes of consumed substances measured during the nights and weekends were similar between the two periods (Fig. 4). As observed within the first 2006-2008 period, a higher proportion of drivers under 35 years old were positive for illicit drugs compared to older drivers, while the number of people positive for a pharmaceutical tended to proportionally increase with age (Fig. 3).

Our study confirms that male drivers tend to consume more drugs than women (11.5 and 14% of positive cases versus 8.3 and 8.5% for the two periods of the study, respectively). During the first period, 1.7% of women and 4.4% of men were positive for an illicit drug, while those numbers reached 6.4 and 7.4% regarding pharmaceuticals. From 2017 to 2020, 5 and 9.1% of men and 3.4 and 5.1% of women were positive for an illicit drug and a pharmaceutical, respectively.

During this period of the study, 7 samples were collected by the police. Among them, 3 were positive for alcohol above the legal cut-off (BrAC > 0.25 mg/L), while 4 were positive for a drug. One was positive for pharmaceuticals (antidepressants and benzodiazepine), and the 3 others were positive for an illicit drug (2 cases of THC and one case of cocaine).

## Discussion

The findings in this study show that in Western Switzerland the prevalence of drugs in random traffic was similar between 2006-2008 and 2017-2020. Considering illicit drugs, a higher proportion of drivers under 35 years old were positive compared to older drivers. This tendency was reinforced during nights and weekends, periods which are considered particularly prone to fatal accidents for this age group. Remarkably, the response to the road safety questionnaire performed during the study also revealed that people have a weak perception of the risk of undergoing police checks while driving after the consumption of a psychoactive substance. This feeling is not surprising, considering that the American Centers for Disease Control and Prevention (CDC) reported that only 1% of the 111 million self-reported alcohol-impaired driving cases were arrested for DUID, which is probably an underestimation [35].

Focusing on the prevalence of psychoactive substances, the results of the study are in line with the prevalence within the general population, with benzodiazepines, antidepressants, antipsychotics and opioids being the most frequently detected pharmaceuticals. The results are more surprising with regard to illicit drugs since cocaine was the most frequently detected substance with a higher prevalence than cannabis. According to the Swiss Office for the Coordination of Addiction Facilities 2022 report, most cocaine users consume within a casual and festive context, mainly in the evening and on the weekends [12]. On the other hand, cannabis is mainly consumed at home or in a private context. In addition, these results must be put into perspective with the study’s design. Indeed, the drivers were invited to participate on an anonymous and voluntary basis only if no consumption was previously suspected by the police. This probably leads to an underestimation of the illicit drugs prevalence since individuals might tend to hide illicit and stigmatizing behaviours [11]. Even considering these biases, which tend to decrease the probability of positive cases, more than 10% of the collected samples were positive for a substance that might impair the driving abilities in both periods of the study.

Despite the decrease in mortality observed on Swiss roads in the last 10 years, the study results demonstrate that the consumption of illicit drugs may be considered as stable, especially during nights and weekends, where the risk of mortal accidents is the highest for young drivers [35, 36]. These results highlight the difficulty of bringing to light psychoactive substance consumption other than alcohol and cannabis based on visual and subjective perceptions. This aspect is also supported when comparing the prevalence of substances in studies based on suspected and unsuspected drivers [15, 20, 21]. In studies performed on suspected drivers, alcohol and cannabis represent most of the positive cases by far, while in the present study cocaine and benzodiazepines have the highest prevalence regarding drugs. Another point that suggests that sample collection performed on suspected drivers is biased is the difference in drivers’ average age. In studies performed on suspected drivers previously in Switzerland, the average age was approximately 30 (27 ± 7 [21], 28 ± 10 [22] and 31 ± 12 [15]), while in both periods of this study, the average age was 41 ± 15 between 2006 and 2008 and 39 ± 15 between 2017 and 2020. These results highlight the targeted aspect of suspicion-based sample collection.

Globally, DUID is an important cause of road accidents. In America, the National Highway Traffic Safety Administration (NHTSA) states that alcohol-impaired drivers accounted for 28% of all traffic-related deaths in 2014 and that psychoactive drugs were involved in 16% of motor vehicle crashes [36]. In the 2006-2008 period of the study, weekends and nights were identified as particularly at-risk time periods regarding DUID (Figs. 2B and C). The results are in accordance with the statistics provided by the NHSTA and the Insurance Institute for Highway Safety (IIHS), showing an increased risk of fatal crashes on Fridays, Sundays and especially on Saturdays [37]. Moreover, the CDC has identified night-time driving as particularly prone to accidents, especially for young drivers, and they state DUID as an increased risk factor [35]. In the present study, the fact that young drivers (under 35 years old) are more prone to drive under the influence of an illicit drug (Fig. 3), especially during nights and weekends (Fig. 2D), was confirmed. Remarkably, the comparison of both time periods of the study suggests that there is no decrease in psychoactive substance consumption in the last 10 years, especially when focusing on at-risk time periods, even if the total number of fatal accidents has diminished [6]. This observation highlights that prevention regarding DUID may still be improved to decrease the number of drivers under the influence.

This study shows that there is a need to increase the efficiency of both prevention and deterrence and that unbiased systematic sampling strategies might be an interesting asset to this end. With the development of analytical tools and strategies, especially regarding quantitative analyses [28, 38, 39] on microsamples, DBS sampling presents new opportunities from this perspective. Indeed, both qualitative and quantitative analyses can be performed directly on blood samples, allowing direct toxicological interpretation. During the 2017-2020 period of our study, the introduction of a high-resolution MS (HRMS) analyser brought new opportunities by enlarging the number of substances of interest from 103 to more than 1000 from fingertip blood sampling.

## Limitations

The extent to which the prevalence of drugs measured in this study can be extrapolated to the whole population of drivers in western Switzerland might suffer from a few limitations. For instance, when comparing the three different road types (Highway, Main Road and City), it does not consider the underlying fact that different numbers of samples representing each category were collected during weekends. This aspect is especially true regarding the 2017 to 2020 study on DBS samples, where the total number of analysed samples (N) is limited.

Second, even if the number of people refusing the sampling is limited (18% regarding OF and 8% regarding DBS), it might be reasonable to assume that drivers who had consumed drugs were less likely to participate in the study, even though it was clear that the study was anonymous and dissociated from the police procedure. Moreover, it has been stated in previous studies regarding alcohol consumption in Belgium that if a control lasted for more than one hour at the same road site, the number of positive samples decreased [40, 41]. This is probably explained by the fact that drivers passing the control site then warned other drivers. Almost all the controls performed within this study lasted for 90 minutes to collect enough samples, which might result in some bias. In addition, people suspected by the police were not transferred to the research site. If the information regarding those cases were ultimately sent by the police, this leads to an underestimation of the positive samples considered in the present study results.

Third, the use of different biological matrices between the two sampling periods probably impacted the results. Indeed, blood is the matrix of reference for toxicological interpretation, including DUID evaluation, since there is a good correlation between the drug concentration and pharmacological effects [42]. Various studies have been conducted to find a correlation between OF and blood or plasma drug concentrations [43–45]. Unfortunately, there is a large interindividual variation in the concentration ratios between those different matrices [46]. Indeed, there are differences between blood and OF in the way the drugs transfer into the biological matrix [47]. For instance, the trapping of basic compounds such as amphetamines is favoured into OF, while more acidic compounds such as cannabinoids are better trapped in blood [48]. Accordingly, OF concentrations cannot be used to calculate a reciprocal concentration in the blood. These aspects might have led to small differences between the two biological matrices used regarding the detection and identification of some substances.

Last, the positive results to pharmaceuticals should be interpreted cautiously. Indeed, if illicit and many pharmaceuticals might impact the driving abilities, the diversity of chemical properties and pharmacological effects do not allow a general statement on these abilities [17]. Moreover, in this study context, the available information did not allow to differentiate a pharmaceutical misuse or a recreational use from a consumption under prescription. It should also be noted that the second period of this study was limited to qualitative analyses even though thresholds were used in adequacy with legal cut-offs or therapeutic ranges to avoid false-positive results. Indeed, among other parameters, limits of identification associated with the intensity threshold were evaluated during the development and validation of the analytical procedures. However, the presence of a substance in either blood or OF does not necessarily imply that this consumption resulted in a driving impairment. These limitations notably associated with the study design tend to suggest that the results presented herein are underestimating the prevalence of drug use while driving and that a significant investment is required in terms of prevention and deterrent measures. The use of systematic sampling strategies associated with the development of proper on-site sampling tools provides an interesting asset to this end.

## Conclusion

In 2014, 38 million people above 16 years old were reported to drive under the influence of either alcohol, drugs or both in the USA [4]. In light of the risk of driving under the influence of drugs on road accidents, our study assessed the prevalence of drug consumption in drivers of Western Switzerland between 2006 and 2008 and between 2017 and 2020. Interestingly, drivers reported a weak probability of being positively controlled by police, whereas a high percentage of positivity to psychoactive substances was measured in the biological fluids of these drivers. This positivity was particularly important for drivers below 35 years old during night and week-end periods. Despite the reduction in fatal accidents in Switzerland over the last 10 years, the consumption of drugs during driving was stable when comparing the 2006-2008 and 2017-2020 periods.

Even though the study was performed on a voluntary basis following a preliminary police control, the important number of positive cases should encourage biofluid sampling strategies. Globally, these results reveal the need for better prevention and deterrence of DUID that could potentially contribute to reducing the risk of fatal accidents.

## Supplementary Information

**Additional file 1.**

**Additional file 2.**

**Additional file 3.**

## Data Availability

All data generated or analysed during this study are included in the published article (and its Additional file 2: supplementary information file named RawDataSupplementary).

## Abbreviations

DRUID project: Driving Under the Influence of Drug, Alcohol and Medicine project
DUID: Driving Under the Influence of Drugs
OF: Oral Fluids
DBS: Dried Blood Spot
LC: Liquid Chromatography
MS/MS: Tandem Mass Spectrometry
BrAC: Breath Alcohol Concentration
MRM: Multiple Reaction Monitoring
HS: Headspace
GC: Gas Chromatography
FID: Flame Ionization Detector
CDC: Centers for Disease Control and Prevention
NHTSA: National Highway Traffic Safety Administration
IIHS: Institute for Highway Safety
HRMS: High Resolution Mass Spectrometry
