# Supplementary material for: Randomly controlled drivers using minimally invasive sampling: assessment of drug prevalence in Western Switzerland over two time periods
Source: BMC Public Health. 2022 Dec 28;22:2446. doi: 10.1186/s12889-022-14883-2 (PMC9795657; doi:10.1186/s12889-022-14883-2)
Supplement: Supplementary file 1 — Additional file 1. [file 12889_2022_14883_MOESM1_ESM.pdf]

Supplementary Table 1 : List of the 103 targeted substances during both parts of the study and their associated crude prevalence by molecule and by class of molecules. One sample can be positive to several substances within the same class of molecule. Substances are categorized into 4 classes according to the DRUID 6th framework classification programme (Class I = minor influence on fitness to drive; Class II = moderate influence on fitness to drive; Class III = sever influence on fitness to drive).

Class of molecule	Substance	Risk class (Druid project)	Prevalence 2006-2008	Prevalence by class 2006-2008	Prevalence 2017-2020	Prevalence by class 2017-2020
**Benzodiazepines and z-drugs**	Alprazolam	Class III	3	29	1	8
	Bromzepam	Class III	5		0	
	Clobazam	Class II	1		3	
	Clonazepam	Class II or III (Administration)	0		1	
	Diazepam	Class III	1		0	
	Flunitrazepam	Class III	0		0	
	Flurazepam	Class III	1		0	
	Ketazolam	NE	0		0	
	Lorazepam	Class III	3		0	
	Lormetazepam	Class III	0		0	
	Midazolam	Class III	0		0	
	Nitrazepam	Class III	0		0	
	Nordiazepam	NE	5		2	
	Oxazepam	Class III	2		0	
	Prazepam	Class III	0		0	
	Temazepam	Class III	0		0	
	Triazolam	Class III	0		0	
	Zaleplon	Class III or I (after 12h)	0		0	
	Zolpidem	Class III or II (after 8h)	12		3	
	Zopiclone	Class III	1		1	
**Antidepressants**	Amitriptyline	Class III	2	34	0	2
	Citalopram	Class I	12		1	
	Clomipramine	Class II	1		0	
	Dibenzepin	Class III	0		0	
	Doxepin	Clas III	0		0	
	Fluoxetine	Class I	0		0	
	Fluvoxamine	Class I	0		0	
	Imipramine	Class II	0		0	
	Maprotiline	Class II or III (Administration)	0		0	
	Melitracen	NE	3		0	
	Mianserin	Class III	0		0	
	Mirtazapine	Class III	5		0	
	Moclobemide	Class II	0		0	
	Nortriptyline	Class II	3		0	
	Opipramol	NE	1		0	
	Paroxetine	Class I	6		1	
	Reboxetine	Class I	0		0	
	Sertralin	Class I	0		1	
	Trazadone	Class III	1		0	
	Trimipramine	Class II	1		0	
	Venlafaxine	Class II	3		0	
**Amphetamines Stimulants**	Amphetamine	Illicit Drug	1	1	0	1
	Cathine	NE	0		0	
	Ephedrine	Class I	0		0	
	MDA	Illicit Drug	0		0	
	MDE	Illicit Drug	0		0	
	MDMA	Illicit Drug	0		1	
	Methamphetamine	Illicit Drug	0		0	
	Methylphenidate	Class II	0		0	
	Modafinil	Class II	0		0	
**Barbiturates**	Butalbital	NE	0	0	0	0
	Pentobarbital	NE	0		0	
	Phenobarbital	Class III	0		0	
	Secobarbital	NE	0		0	
	Thiopental	Class III	0		0	
**Neuroleptics**	Amisulpride	Class II	2	13	1	1
	Aripiprazole	Class II or III (Administration)	0		0	
	Chlorpromazine	Class III	0		0	
	Chlorprothixen	NE	0		0	

	Clotiapine	Class II or III (Administration)	1		0	
	Clozapine	Class III	4		0	
	Flupentixol	Class II or III (Administration)	0		0	
	Fluphenazine	Class II	0		0	
	Haloperidol	Class II or III (Administration)	0		0	
	Levomepromazine	Class III	1		0	
	Olanzapine	Class II or III (Administration)	0		0	
	Penfluridol	Class II	0		0	
	Perphenazine	Class II	0		0	
	Pimpamperone	Class II	1		0	
	Promazine	Class III	0		0	
	Quetiapine	Class II	2		0	
	Risperidone	Class II or III (Administration)	0		0	
	Sulpiride	Class II	2		0	
	Tiapride	Class II	0		0	
	Zuclopenthixol	Class II or III (Administration)	0		0	
**Opioids**	6-MAM	Illicit Drug	1	16	2	5
	Buprenorphine	Class II or III (Administration)	1		0	
	Codeine	Class I or II (Dose)	2		2	
	Dextrometorphan	Class I	5		0	
	Ethylmorphine	Class III	0		0	
	Hydrocodone	Class II	0		0	
	Hydromorphone	Class III or II (Steady state)	0		0	
	Methadone	Class II or III (Administration)	5		0	
	Morphine	Class III or II (Steady state)	0		2	
	Nalbuphine	NE	0		0	
	Oxycodone	Class III or II (Steady state)	1		1	
	Pholcodine	Class II	0		0	
	Tramadol	Class III	3		2	
**Cocaine**	Cocaine	Illicit Drug	30	30	8	8
	Benzoylecgonine	Illicit Drug	8		8	
	Ethylcocaine	Illicit Drug	0		2	
**Cannabinoids**	THC	Illicit Drug	5	6	-	6
	11-OH-THC	Illicit Drug	0		0	
	THCCOOH	Illicit Drug	0		6	
**Other drugs**	Buspirone	Class I	3		0	
	Carbamazepine	Class II	1		0	
	Clomethiazol	Class I	0		0	
	Diphenhydramine	Class III	1		0	
	Hydroxyzine	Class II	1		0	
	Lamotrigine	Class II	0		1	
	Levetiracetam	Class II	0		0	
	Meprobamate	NE	0		0	
	Oxcarbazepine	Class II	2		0	
