# Supplementary material for: Randomly controlled drivers using minimally invasive sampling: assessment of drug prevalence in Western Switzerland over two time periods
Source: BMC Public Health. 2022 Dec 28;22:2446. doi: 10.1186/s12889-022-14883-2 (PMC9795657; doi:10.1186/s12889-022-14883-2)
Supplement: Supplementary file 3 — Additional file 3. [file 12889_2022_14883_MOESM3_ESM.pptx]

## Slide 1
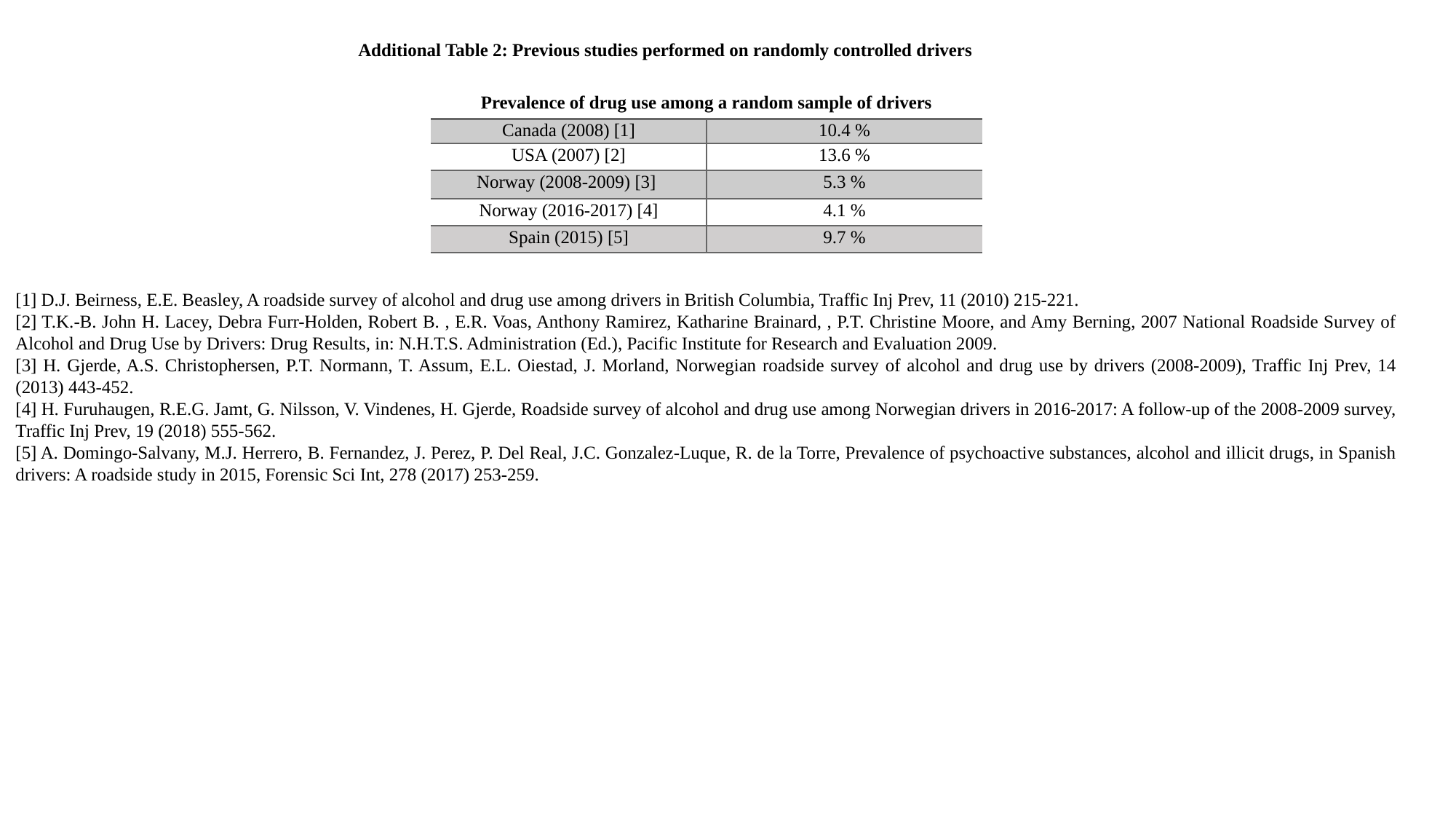

Additional Table 2: Previous studies performed on randomly controlled drivers
| Prevalence of drug use among a random sample of drivers | |
| --- | --- |
| Canada (2008) [1] | 10.4 % |
| USA (2007) [2] | 13.6 % |
| Norway (2008-2009) [3] | 5.3 % |
| Norway (2016-2017) [4] | 4.1 % |
| Spain (2015) [5] | 9.7 % |
[1] D.J. Beirness, E.E. Beasley, A roadside survey of alcohol and drug use among drivers in British Columbia, Traffic Inj Prev, 11 (2010) 215-221.
[2] T.K.-B. John H. Lacey, Debra Furr-Holden, Robert B. , E.R. Voas, Anthony Ramirez, Katharine Brainard, , P.T. Christine Moore, and Amy Berning, 2007 National Roadside Survey of Alcohol and Drug Use by Drivers: Drug Results, in: N.H.T.S. Administration (Ed.), Pacific Institute for Research and Evaluation 2009.
[3] H. Gjerde, A.S. Christophersen, P.T. Normann, T. Assum, E.L. Oiestad, J. Morland, Norwegian roadside survey of alcohol and drug use by drivers (2008-2009), Traffic Inj Prev, 14 (2013) 443-452.
[4] H. Furuhaugen, R.E.G. Jamt, G. Nilsson, V. Vindenes, H. Gjerde, Roadside survey of alcohol and drug use among Norwegian drivers in 2016-2017: A follow-up of the 2008-2009 survey, Traffic Inj Prev, 19 (2018) 555-562.
[5] A. Domingo-Salvany, M.J. Herrero, B. Fernandez, J. Perez, P. Del Real, J.C. Gonzalez-Luque, R. de la Torre, Prevalence of psychoactive substances, alcohol and illicit drugs, in Spanish drivers: A roadside study in 2015, Forensic Sci Int, 278 (2017) 253-259.
